# Frequency of Mutations in the *TPO* Gene in Patients with Congenital Hypothyroidism Due to Dyshormonogenesis in Chile

**DOI:** 10.3390/medicina60071145

**Published:** 2024-07-16

**Authors:** María Clara Arteaga-Jacobo, Ángel Roco-Videla, Claudio Villota Arcos, Patricio González-Hormazábal, Víctor Gonzalo-Castro, María Virginia Pérez-Flores

**Affiliations:** 1 Programa de Genética Humana, Institute of Biomedical Science (ICBM), Facultad de Medicina, Universidad de Chile, Santiago 8380453, Chile; vgcastro@gmail.com; 2 Vicerectoria de Investigación e Innovación, Universidad Arturo Prat, Iquique 1110939, Chile; 3 Facultad de Medicina, Universidad Católica de la Santísima Concepción, Concepción 4030000, Chile; 4 Escuela de Nutrición y Dietética, Facultad de Ciencias de la Salud, Universidad Bernardo O’Higgins, Santiago 8370993, Chile; claudio.villota@ubo.cl; 5 Hospital San Juan de Dios, Santiago 8350488, Chile; mavypf@gmail.com; 6 Hospital Luis Calvo Mackenna, Santiago 7500539, Chile

**Keywords:** congenital hypothyroidism, dyshormonogenesis, thyroid peroxidase

## Abstract

*Background and Objectives*: Congenital thyroid dyshormonogenesis is caused by alterations in the synthesis of thyroid hormones in a newborn. Additionally, 10 to 20% of these cases are hereditary, caused by defects in proteins involved in hormonal synthesis. One of the most common causes is mutations in the thyroid peroxidase (TPO) enzyme gene, an autosomal recessive disease. We aimed to detect mutations of the *TPO* gene in 12 Chilean patients with congenital hypothyroidism due to dyshormonogenesis (CHD) and to characterize these patients clinically and molecularly. *Materials and Methods*: Twelve patients under 20 years of age with CHD, controlled at San Juan de Dios Hospital in Santiago, Chile, were selected according to the inclusion criteria: elevated neonatal TSH, persistent hypothyroidism, and thyroid normotopic by imaging study. Those with deafness, Down syndrome, and central or transient congenital hypothyroidism were excluded. Blood samples were taken for DNA extraction, and the 17 exons and exon–intron junctions of the *TPO* gene were amplified by PCR. The PCR products were sequenced by Sanger. *Results*: Two possibly pathogenic mutations of the *TPO* gene were detected: c.2242G>A (p.Val748Met) and c.1103C>T (p.Pro368Leu). These mutations were detected in 2 of 12 patients (16.6%): 1 was compound heterozygous c.1103C>T/c.2242G>A, and the other was heterozygous for c.2242G>A. In the diagnostic confirmation test, both patients presented diffuse hyper-uptake goiter on thyroid scintigraphy and high TSH in venous blood (>190 uIU/mL). *Conclusions*: The frequency of patients with possibly pathogenic mutations in *TPO* with CHD was 16.6%. Its study would allow for genetic counseling to be offered to the families of affected patients.

## 1. Introduction

Congenital hypothyroidism corresponds to a deficiency of thyroid hormones diagnosed at birth [1]. The classic clinical symptoms and signs of congenital hypothyroidism are usually absent immediately after birth in most infants due to temporary protection from maternal thyroxine during pregnancy. This condition is one of the most common preventable causes of intellectual disability in countries where there are screening programs for newborns. On the other hand, it can produce critical neurological alterations if it is not treated promptly with replacement therapy with synthetic thyroid hormone (T4) in the early stages of a child’s development [2]. Thyroid hormones intervene critically during the development of the central nervous system, influencing migration, differentiation, and neuronal signaling [3].

The worldwide prevalence of congenital hypothyroidism is estimated to be between 1 in 2000 and 1 in 4000 live births. In Chile, this disease has an incidence of 1 in 3163 live newborns, is the most common endocrine pathology in newborns, and is the most frequent cause of preventable intellectual disability [4].

The causes of primary congenital hypothyroidism are varied, and most of them occur in people with no family history of the disease. However, it is estimated that approximately 10 to 20% of cases of primary congenital hypothyroidism are hereditary [5,6]. Within primary congenital hypothyroidism, 85% of cases are due to defects in the development of the thyroid gland or dysembryogenesis, resulting in thyroid absence, hypoplasia, or ectopia. Additionally, 10% to 20% of cases of primary congenital hypothyroidism are caused by thyroid dyshormonogenesis. This percentage is equivalent to an incidence of 1:30,000 newborns in Chile [5,7]. These patients are characterized by having an anatomically normal thyroid gland but its synthesis of thyroid hormones is decreased or completely inhibited. Primary congenital hypothyroidism due to dishormonogenesis (CHD) follows an autosomal recessive inheritance pattern [5,6].

Most patients with CHD carry mutations in genes encoding enzymes that participate in synthesizing thyroid hormones. The genes associated with this disease cause alterations in the synthesis of thyroid hormones, including defects in the transport of iodine to the thyrocyte (*SLC5A5*/*NIS*), defects in the organification of iodine *(TPO*, *DUOX1*, *DUOX2*, *DUOXA2*, *SLC26A4*, and *SLC26A7*), alterations in the synthesis of thyroglobulin (*TG*), or iodotyrosine deiodinase deficiency (*IYD*/*DEHAL1*) [8]. The most common cause of CHD is due to mutations in the *TPO* gene [8], although in East Asia the most common mutated gene is *DUOX2* [9,10]. Exons 8 and 9 present the highest density of the described mutations in *TPO*. These exons code for the catalytic site of the enzyme. Approximately 74% of the described mutations are substitutions, and 26% are deletions and insertions that lead to the production of a truncated protein [11,12,13,14,15]. The prevalence of *TPO* gene mutations in cases of CHD varies depending on the population studied, the study method, and the sample selection criteria. Various studies have been published about the mutations associated with CHD—precisely due to mutations in the *TPO* gene—especially in countries where a neonatal screening program and follow-up of patients with congenital hypothyroidism are carried out [11,12].

The European Society of Pediatric Endocrinology consensus guideline on the screening, diagnosis, and management of congenital hypothyroidism [2] states that “genetic counseling should be performed to relatives of patients with congenital hypothyroidism as a fundamental part of the management, in order to determine the mode of inheritance, the risk of recurrence, the possible associations with other syndromic alterations and the long-term prognosis, especially indicating this attention to pregnant women with a history of having had a child with non-syndromic congenital hypothyroidism due to dyshormonogenesis”. Following this recommendation, the purpose of this work is to initiate the genetic study of patients with CHD in Chile, which will allow us to offer timely genetic counseling to our patients’ families in the future. The present report is the first study on this subject in Chile and one of the first in South America. We aimed to describe (a) the clinical and biochemical characteristics of 12 patients with congenital hypothyroidism due to dyshormonogenesis and (b) to identify the frequency of mutations in the *TPO* gene in a group of patients with CHD to associate the genotype of the patients with clinical and biochemical characteristics.

## 2. Materials and Methods

A retrospective study was designed based on a review of a 20-year follow-up database of all patients with congenital hypothyroidism diagnosed in the National Neonatal Screening Program for Congenital Hypothyroidism at the San Juan de Dios Hospital between 1992 and 2013. Twelve of the 23 selected patients agreed to participate. These twelve patients met the selection criteria outlined below and were contacted and offered voluntary participation in the study.

### 2.1. Inclusion Criteria

(a) Subjects with elevated TSH neonatal levels on filter paper at 72 h of life, following the National Neonatal Screening Program for Congenital Hypothyroidism application at the San Juan de Dios Hospital (Reference Center in Chile). Until 2005, the Program used the radioimmunoassay (IRMA) method to quantify TSH, with a 20 uUI/mL cut-off level. After that year, the analysis was changed to immunofluorescence (DELFIA), with a TSH > 15 uIU/mL suggesting congenital hypothyroidism. Those subjects who had altered TSH screening underwent confirmatory blood tests (TSH > 10 uIU/mL in venous blood). (b) Normotopic (orthotopic) thyroid by ultrasound and/or (99m)Tc-pertechnetate thyroid scintigraphy. (c) Persistent congenital hypothyroidism.

### 2.2. Exclusion Criteria

Deafness (to exclude Pendred’s syndrome, characterized by deafness and congenital hypothyroidism), Down syndrome, central congenital hypothyroidism, and transient congenital hypothyroidism.

This study was approved by the Institutional Review Board of the Faculty of Medicine of the University of Chile and San Juan de Dios Hospital, Santiago, Chile. All the parents of the patients were asked to sign the informed consent and patients over 12 years of age were also asked to sign an informed assent.

### 2.3. Genomic DNA Extraction

A 10 mL volume of peripheral blood was obtained by venipuncture in the forearm, a was collected in a vacuotainer tube with EDTA as anticoagulant. Genomic DNA was extracted from the buffy coat using the method described by Chomczynski et al. [13].

### 2.4. Sequencing of the Coding Region of the TPO Gene

The 17 exons of the *TPO* gene were amplified by PCR, using the primer sequences described by Hashemipour et al. 2012 [14] except Exon2 R: 5′ TGTCATCCAAGGAAAATCAGC 3′, Exon 8.1 F: 5′ ACTGGAGGGGCAGAGAAAC 3′ and R: 5′ GCACGAAAGGCAGGTAGG 3′, and Exon 8.2 F: 5′ CAGCAGATGAACGGGTTGAC 3′ R: 5′ TGTGCAAGTAACTGGGAGAG 3′. The primers covered the coding sequence of these exons and the surrounding intronic sequences (more than 30 bases). The thermal cycler programs and PCR mixtures are described in Table S1. PCR products were subjected to the Sanger sequencing procedure in an ABI 3130xl sequencer (performed by Macrogen, Seoul, Republic of Korea). Finally, the ChromasPro version 1.7.6 program was used to analyze the electrophoretograms. We followed the recommendations of HUGO to describe the mutations, using NM_000547 as the reference cDNA sequence of *TPO*.

### 2.5. Methods for Analyzing the Functional Significance of the Nucleotide Changes

A map of the functional domains of the TPO protein was created using SMART v4.1 (http://smart.embl-heidelberg.de/) by submitting the amino acid sequence from ENSP00000318820. Missense changes were analyzed using PolyPhen 2.2.3 r408, SIFT, and PRALINE v2A programs. PolyPhen 2.2.3r408 (http://genetics.bwh.harvard.edu/pph2/) predicts the possible impact of an amino acid substitution on the structure and function of a human protein using direct physicochemical and comparative considerations. The protein sequence for PolyPhen 2.2.3r408 prediction was ENSP00000318820. The SIFT program (http://sift.bii.a-star.edu.sg/) predicts if an amino acid substitution affects protein function based on sequence homology between species and the physicochemical properties of the amino acid. This program provides a score ranging from 0 to 1, indicating harmful substitution if it is close to 0. The amino acid sequence ENSP00000318820 was used for SIFT analyses with by-default settings. In addition, a comparative scheme of the conservation of amino acids among different species was created using the multiple alignment analysis program PRALINE v2A (http://www.ibi.vu.nl/programs/pralinewww), which integrates data from homology investigations between species and secondary structure data. This application provides a score from 1 to 10, with 10 indicating that amino acids are highly conserved between different species. Possible alterations in the splicing sites of synonymous changes were analyzed using SpliceAI lookup (https://spliceailookup.broadinstitute.org/) with by-default settings. All the analyses were performed on 6 June 2024.

## 3. Results

### 3.1. Populations

The study group included 12 unrelated subjects (six women and six men, age < 20 years at the time of the study) belonging to the National Neonatal Screening Program for Congenital Hypothyroidism at the San Juan de Dios Hospital. Table 1 describes the main characteristics of the patients.

### 3.2. Nucleotide Changes

Table 2 shows the nucleotide variations found in the sample and their allele frequency according to the Latino (amr) gnomAD database. The variants c.-125G>A, c.-80A>G, c.12C>G, c.769G>T, c.1193G>C, c.1117G>T, c.1998C >T, c.2145C> T, c.2173A>C, and c.2540T>C present allelic frequencies higher than 0.3, and some are described as polymorphisms in central databases such as OMIM and dbSNP, so they were not considered candidates to be pathogenic. Therefore, the variants c.69C>T, c.1103C>T, c.1449C>T, and c.2242G>A were analyzed as possible candidates to be pathogenic because they are described at a low allelic frequency in several databases or have not been described before. These nucleotide changes were found in three patients: patient 5, who is a compound heterozygous for the c.1103C>T and c.2242G>A mutations; patient 8, who is a simple heterozygous for the c.2242G>A mutation; and patient 10, who is simple heterozygous for the c.1449C>T mutation. Electrophoretograms corresponding to patients 5, 8, and 10 are shown in Figure S1.

### 3.3. In Silico Analysis

**Figure 1:**
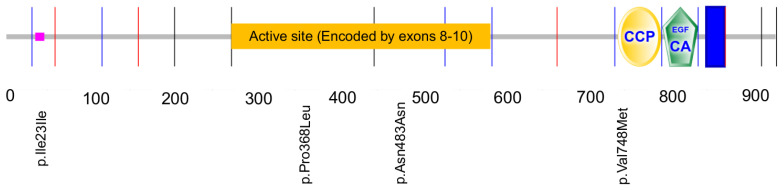
Location of the mutations in the TPO protein. The scheme of functional sites was obtained by the SMART V4.1 program. The active site is also depicted. Vertical lines indicate the exon–exon boundaries, starting from the first coding exon (corresponding to exon 2).

SMART V4.1 software (http://smart.embl-heidelberg.de/) was used to locate the mutations in the different domains of the TPO protein (Figure 1). The two missense mutations (p.Pro368Leu and p.Val748Met) are at the active site and CCP domain, respectively.

**Figure 2:**
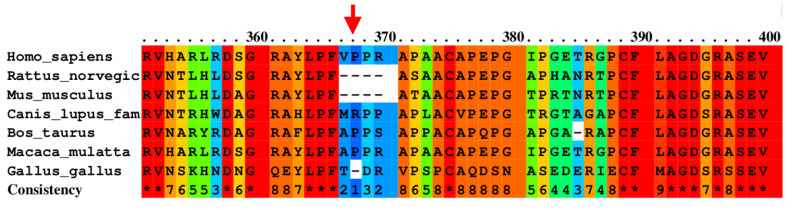
Conservation between different species for the amino acid change of proline to leucine at position 368. The arrow indicates the position of the amino acid. Multiple alignments were obtained by the PRALINE program. Asterisks indicate fully conserved residues.

According to SpliceAI, we propose that the c.69 C>T and c.1449C>T mutations do not modify the splice site. The SIFT 4G software predicts that c.1103C>T is tolerated (SIFT score = 0.26). This variant changes proline to leucine at position 368, which is not conserved between different species (Figure 2). When analyzing this same variant with the PolyPhen 2 program, it is estimated that it is possibly damaging. Taken together, this missense mutation could have a functional effect on the TPO protein.

**Figure 3:**
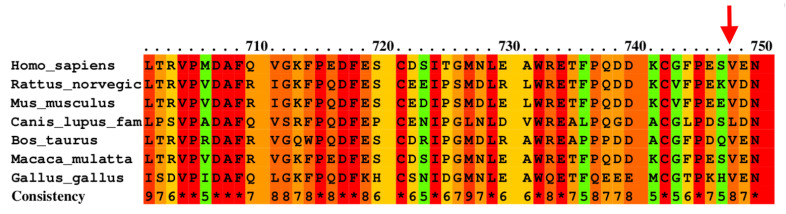
Conservation between different species of the amino acid changed from valine to methionine at position 748. The arrow indicates the position of the amino acid. Multiple alignments were obtained by the PRALINE program. Asterisks indicate fully conserved residues.

The nucleotide variation c.2242G>A was analyzed with PolyPhen 2, resulting in an indication of possibly damaging action, and SIFT software predicted that it affects protein function. Both approaches propose that the amino acid substitution would have a functional effect. Moreover, the nucleotide variation c.2242G>A produces a change from valine to methionine at position 748, which is highly conserved between different species, further supporting its pathogenicity (Figure 3). Taken together, the nucleotide variations c.1103C>T and c.2242G>A met the criteria to be considered possibly pathogenic. Table 3 summarizes the analyses of the c.69C>T, c.1449C>T, c.1103C>T, and c.2242G>A variants.

### 3.4. Clinical and Biochemical Characteristics of Two Carriers of c.1103C>T and c.2242G>A TPO Mutations

Both carriers (patients 5 and 8) presented severe congenital hypothyroidism with diffuse goiter with hyper-uptake on the thyroid scintigram and elevated levels of TSH in venous blood during the examination of diagnostic confirmation (>190 uIU/mL) at the time of neonatal diagnosis. However, these characteristics would not be exclusive to both, since patients 7, 9, 10, 11, and 12 also presented diffuse goiter with hyper-uptake on thyroid scintigram. Patient 10 also presented TSH levels > 190 uIU/mL in venous blood in the confirmatory study (Table 1). The psychomotor development of patients 5 and 8 was normal, which is expected given that both received the appropriate hormonal treatment promptly. Images of the thyroid scintigrams of both patients are shown in Figure S2.

## 4. Discussion

Two nucleotide changes in *TPO*, found in two unrelated patients with CHD, were predicted to have a functional effect on the encoded protein. c.1103C>T changes from proline to leucine at position 368, and the nucleotide variation c.2242G>A produces a change from valine to methionine at position 748. These possibly pathogenic nucleotide changes were found in two patients: patient 5, who is compound heterozygous for the mutations c.1103C>T and c.2242G>A, and patient 8, who is simple heterozygous for c.2242G>A.

The c.2242G>A substitution (rs28991292, p.Val748Met) is described in ClinVar as a variant of uncertain significance and currently has conflicting pathogenicity classifications. According to the American College of Medical Genetics (ACMG) recommendations, it has been reclassified as likely pathogenic [15]. We propose that it is a pathogenic variant, considering its low allelic frequency, location in the TPO protein, and the in silico results. It should be noted that a study recently carried out in Argentina in 2022 [15] found the same variant in four patients with congenital hypothyroidism. They also carried out an in silico prediction of the functional studies of the mutation, which concluded that it would be a possibly pathogenic mutation that produces severe changes on the molecular surface, inducing loss of TPO activity [15]. In the Argentinian study, it was also the most frequent pathogenic mutation found in their series of patients. Zhang et al. [16] estimated an in vitro a residual activity of 63% of the TPO enzyme encoded by the variant allele (748Met), giving an antecedent in favor of the pathogenicity of this mutation.

The c.1103C>T (rs1252306521, p.Pro368Leu) variant has been described as a rare allele. This variant is located at exon 8, which encodes the catalytic site of the enzyme. No functional studies of the mutation have been published, and, to the best of our knowledge, this variant has not been reported in other patients with congenital hypothyroidism. The in silico predictions suggest that this mutation could affect the functionality of the TPO enzyme. According to Garcia et al. [17] and Wilcox et al. [18], the use of in silico predictions is a reliable tool to assign the level of pathogenicity established by the American College of Medical Genetics (ACMG) [19]. Nevertheless, this finding deserves further in vitro analysis to evaluate a loss of enzyme activity.

CHD has an autosomal recessive inheritance. The phenotype of patient 8, heterozygous for c.2242G>A and no mutation detected in the other allele, could be explained by the presence of a second mutation not detected in the screening used in this study, as would be the case of the presence of large deletions and insertions, or mutations in regulatory elements. On the other hand, according to international studies, the presence of patients with CHD who are simple heterozygotes for a *TPO* mutation is not uncommon. It could be due to monoallelic expression of the mutated gene [7,15,20,21].

It is estimated that 17% of patients with the typical phenotype have only one allele of the mutated *TPO* gene [20]. The first to study the mechanism of this phenomenon were Fugazzola et al. [20]. They reported a family of both healthy parents, not consanguineous, with three sons with CHD and total iodide organification defects (TIOD). In DNA samples of leukocytes, fibroblasts, and thyroid tissue cells from the healthy father and the three affected children, the p.R693W mutation was found in a simple heterozygous state. The mRNA study in the thyroid tissue of the affected patients demonstrated the absence of mRNA transcripts of the normal maternal allele in the three affected children. The performed analysis ruled out the possibility that these patients presented large deletions or insertions in the 2p25 region of the maternal chromosome or mutations in the promoter of the *TPO* gene. A methylation study of the regulatory region of the *TPO* gene was also carried out, which ruled out a possible alteration in maternal imprinting. Finally, they propose the presence of mutations in distant regions of the *TPO* gene, such as deep intronic regions, leading to an accelerated degradation of the mRNA. In a study carried out in Argentina in four patients who were simple heterozygotes for a *TPO* mutation, the presence of a second mutation in some other region of the same gene was ruled out, and it was suggested that they probably present a monoallelic expression of the mutated gene [21]. In Brazil, Neves et al. [22] described a patient affected by CHD that was simple heterozygous for a mutation in *TPO*. The patient did not present any other mutations, neither in the promoter region of this gene or in the *DUOX2* or *DUOXA2* genes. The authors suggest a possible monoallelic expression of the mutated *TPO* gene. On the other hand, the finding of a homozygous deletion of exons 11 to 15 in the *TPO* gene in patients with congenital hypothyroidism in a Pakistani family has been reported [23].

We found a frequency of 16.6% (2/12) of patients with non-polymorphic nucleotide changes in *TPO*. Compared to similar studies in other countries, our finding is lower than those reported by Rodrigues et al. (23%) [24] and Belforte et al. (30%) [25], both using Sanger sequencing. In these studies, patients with a family history of congenital hypothyroidism were included; this was not a selection criterion in our study. The difference with the prevalence found in Wu et al. [12] is surprisingly high, close to 100%, if compared to what was published by Santos et al. [11] and Cangül et al. [7]. This could be because these authors used more restrictive selection criteria, such as the presence of goiter on thyroid scintigraphy and perchlorate testing compatible with an organification defect, which would increase the probability of finding patients with a *TPO* gene mutation in the selected sample. On the other hand, some studies report lower prevalences than those found in our study: 2.4% in Hashemipour et al. [14] and 13% in Ambrugger et al. [26]. This could be explained by the different methodologies used in the molecular study. Thus, in the study by Hashemipour et al., the authors indicate that few cases with the mutation were detected, possibly due to the mutation analysis technique used (SSCP), which has a lower sensitivity than direct sequencing. On the other hand, in the study by Ambrugger et al., only some exons of the *TPO* gene were analyzed, not the entire gene.

From a clinical view, the phenotype of the two patients with CHD carrying possibly pathogenic changes in the *TPO* presents higher levels of TSH in venous blood at the time of diagnosis compared to patients who did not present these nucleotide variations. This is consistent with the expected phenotype, since the enzyme thyroid peroxidase plays an essential role in thyroid hormonogenesis by carrying out oxidation, organification, and coupling reactions, fundamental stages for the synthesis of thyroid hormones T3 and T4. The above findings allow us to differentiate this type of dyshormonogenesis from that produced by a mutation of the gene encoding the TSH receptor or the NIS cotransporter, in which there is hypo-uptake of the radioisotope in the thyroid scintigram [2,6,27]. On the other hand, having high levels of TSH favors rapid uptake of radioiodine [28]. However, patients 7, 9, 10, 11, and 12 also presented diffuse hyperenhancement goiter. This could be explained by the fact that these patients could have mutations in other genes affecting organification, such as DUOX2, TG, DUOXA2, and SLC26A4. Physiologically, in healthy full-term newborns, immediately after birth, there is a rise in TSH levels in response to hypothermia related to the change to an extrauterine environment, which leads to an increase in T3 and T4 levels that remains until approximately the seventh day of life, decreasing after that. Therefore, it is expected that a defect in the action of this thyroid peroxidase enzyme will translate into a severe and permanent deficiency of thyroid hormones, presenting clinically with low plasma levels of these hormones and, as a response, higher levels of TSH.

The above finding was found by Narumi et al. in a group of 14 Japanese patients with CHD [29]. Seven causal genes for thyroid dyshormonogenesis were studied in these cases, including *TPO*. It was shown that patients with mutations in the TPO gene presented higher levels of TSH in venous samples at diagnosis compared to patients with mutations in the other genes studied, in which a severe deficiency in the synthesis of the thyroid hormones does not necessarily occur. Furthermore, in our study, all patients with possibly disease-associated mutations in the *TPO* gene had diffuse goiter with hyperenhancement on thyroid scintigraphy. This fact can be explained, as the defect produced by a mutation in *TPO* would affect the stages after the entry into the thyrocyte of the radioisotope used in the scintigram. The above allows us to differentiate this type of dyshormonogenesis from that produced by a mutation of the gene that codes for the TSH receptor or the NIS cotransporter, in which there is hypo-uptake of the radioisotope in the thyroid scintigram [2,6,27]. On the other hand, high levels of TSH favor a rapid uptake of radioiodine [28].

In this study, patients were not tested for perchlorate, as it was not included in the National Program. Had this been carried out, it would have been possible to better define the candidate patients as presenting a mutation in the *TPO* gene, since this test allows us to better determine patients with dyshormonogenesis due to defects in the organization characteristic of patients with defects in the thyroid peroxidase enzyme. Nevertheless, other genes, different from TPO, are involved in this stage of thyroid hormone synthesis, such as *DUOX2*, *TG*, *DUOXA2*, and *SLC26A4*.

## 5. Conclusions

The variants c.1103C>T and c.2242G>A showed evidence that makes them candidates for causing congenital hypothyroidism. Our study is the first to propose the *TPO* gene variant c.1103C>T as a pathogenic mutation. It is necessary to expand the genetic study of patients with CHD to other populations in Latin America, as this would allow the offer of genetic counseling to affected families and the development of personalized therapies.

## Figures and Tables

**Table 1:** Clinical, anatomical, and biochemical characteristics of the included patients.

Patient	Sex	Age	TSH Screening (µIU/mL)	Venous Blood TSH at Diagnosis (µIU/mL)	Total T4 at Diagnosis (µg/dl)	Free T4 at Diagnosis (ng/dl)	T3 at Diagnosis (ng/dl)	Thyroid Ultrasound	Thyroid Scintigram
1	F	3y	35.00	10.88	9.30	1.16	N.D.	Normotopic	Lower contrast
2	F	2y	43.50	10.81	13.10	1.12	N.D.	Normotopic	Lower contrast and pickup
3	F	9y	82.90	112.90	8.60	1.40	N.D.	Normotopic	Lower contrast
4	M	2y	24.70	39.63	13.30	1.54	N.D.	Normotopic	Contrast asymmetry of both lobes greater thyroid on the right
5	M	1y	37.80	196.38	0.10	0.39	N.D.	Normotopic	Diffuse hyperenhancing goiter
6	M	11y	63.00	20.40	9.30	N.D.	102.00	Normotopic	Lower contrast
7	F	6y	59.80	57.60	10.20	1.70	N.D.	Normotopic	Diffuse hyperenhancing goiter
8	F	12y	210.00	337.76	6.90	0.59	N.D.	Normotopic	Diffuse hyperenhancing goiter
9	M	12y	712.10	148.10	2.56	N.D.	51.00	Normotopic	Diffuse hyperenhancing goiter
10	F	14y	297.90	221.00	2.40	N.D.	114.00	Normotopic	Diffuse hyperenhancing goiter
11	M	15y	44.10	36.50	N.D.	N.D.	N.D.	Normotopic	Diffuse hyperenhancing goiter
12	M	17y	90.80	28.80	8.30	N.D.	130.00	Normotopic	Diffuse hyperenhancing goiter

F: female, M: male, y: years. N.D.: Not determined.

**Table 2:** Description of the nucleotide variations found.

Nucleotide Variations	dbSNP	Exon	Effect on Protein	Allele Frequency ^(2)^
c.-125G>A	rs2071402	Pr	NE	0.51
c.-80A>G	rs2071403	1	NE	0.70
c.12C>G	rs9678281	2	p.Leu16Leu	0.41
c.69C>T	NC_000002.11:$$$$$g.1418249C>T ^(1)^	2	p.Ile23Ile	ND
c.769G>T	rs4927611	7	p.Ala269Ser	0.38
c.1103C>T	rs1252306521	8	p.Pro368Leu	^(3)^
c.1193G>C	rs2175977	8	p.Ser398Thr	0.65
c.1117G>T	rs2280132	8	p.Ala373Ser	0.50
c.1449C>T	rs1226050353	9	p.Asn483Asn	^(4)^
c.1998C>T	rs1126797	11	p.Asp666Asp	0.41
c.2145C>T	rs732608	12	p.Pro658Pro	0.45
c.2173A>C	rs732609	12	p.Thr725Pro	0.46
c.2242G>A	rs28991292	13	p.Val748Met	0.03
c.2540T>C	rs1126799	15	p.Val847Ala	0.61

NE: No effect. ND: Not described. Pr: Promoter. ^(1)^ Genomic coordinates according to GRCh 37 genome ensemble, ^(2)^ according to in Latino (amr) gnomAD database, ^(3)^ one of 3446 alleles described only in finish (fin) gnomAD database, ^(4)^ two of 34590 alleles described only in Latino (amr) gnomAD database.

**Table 3:** Analysis of the pathogenicity criteria of the non-polymorphic nucleotide changes in the *TPO* gene found in the patients.

Patient	Nucleotide Variations	Effect on Protein	Conservation	In Silico Analysis
5	c.69C>T	p.Ile23Ile	NE	SpliceAI. Not new splicing site
10	c.1449C>T	p.Asn483Asn	NE	SpliceAI. Not new splicing site
5	c.1103C>T	p.Pro368Leu	Low	Polyphen2: Possibly damaging (91%). SIFT: Tolerated (0.31)
5 and 8	c.2242G>A	p.Val748Met	High	Polyphen2: Possibly damaging (99%). SIFT: affects protein function (0.02)

NE: Not evaluated.

## Data Availability

The data presented in this study are available on request from the corresponding author.

## Supplementary Materials

The following supporting information can be downloaded at: https://www.mdpi.com/article/10.3390/medicina60071145/s1, Figure S1: Electrophoretograms of Sanger sequencing for the mutations found in patients 5, 8 and 10; Figure S2: Tc-pertechnetate thyroid scintigraphy of patient 5; Table S1: PCR conditions to amplify each exon.
