# Supplementary material for: Frequency of Mutations in the TPO Gene in Patients with Congenital Hypothyroidism Due to Dyshormonogenesis in Chile
Source: Medicina (Kaunas). 2024 Jul 16;60(7):1145. doi: 10.3390/medicina60071145 (PMC11279067; doi:10.3390/medicina60071145)
Supplement: Supplementary file 1 [file medicina-60-01145-s001.zip › medicina-2995511-supplementary/Figure S1.pdf]

Figure S1. Electrophoretograms of Sanger sequencing for the mutations found in patients 5, 8 and 10.

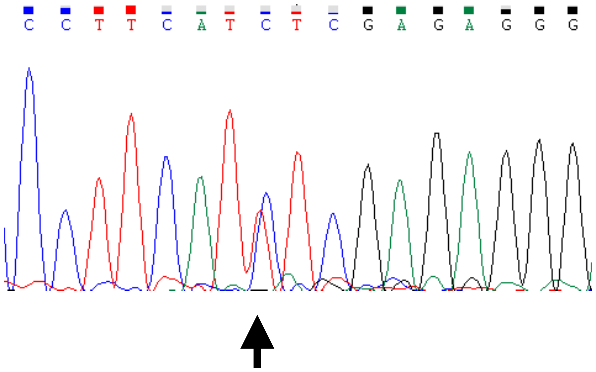

c.69C>T
(Patient 5)

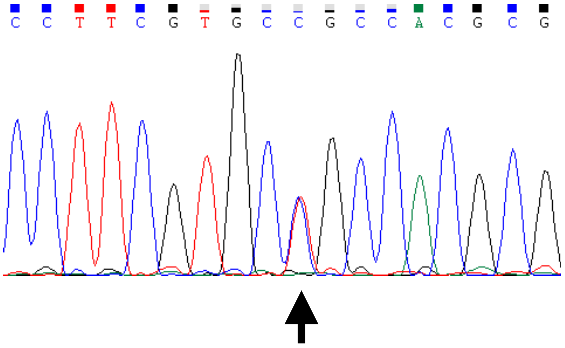

c.1103C>T
(Patient 5)

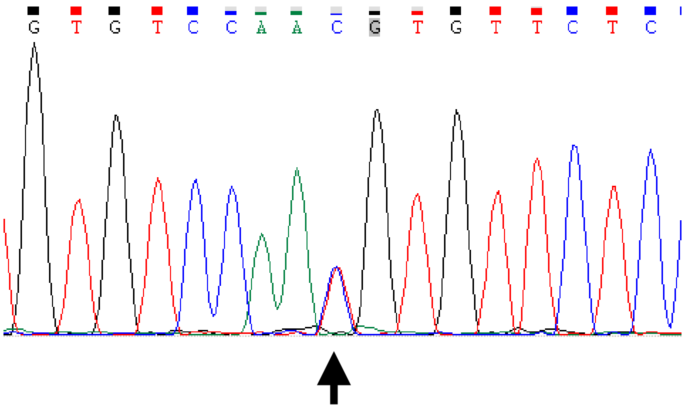

c.1449C>T
Patient 10

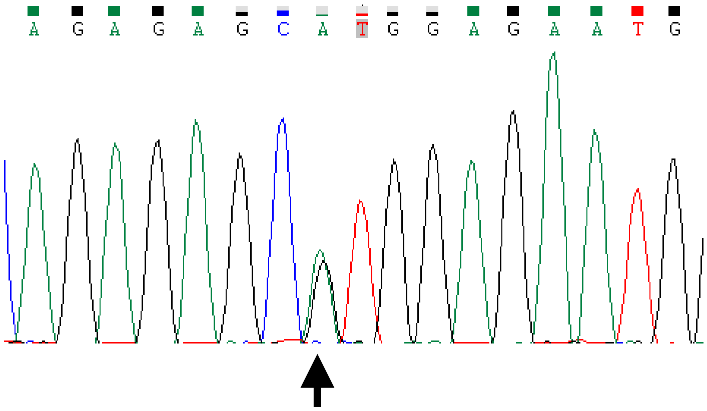

c.2242G>A
(Patient 5)

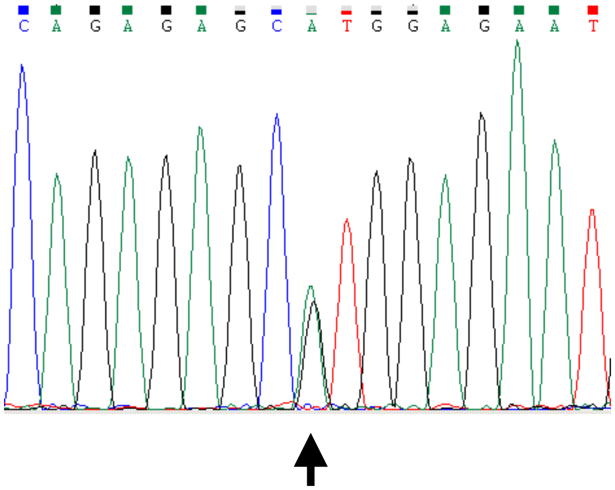

c.2242G>A
(Patient 8)
