# Supplementary material for: Frequency of Mutations in the TPO Gene in Patients with Congenital Hypothyroidism Due to Dyshormonogenesis in Chile
Source: Medicina (Kaunas). 2024 Jul 16;60(7):1145. doi: 10.3390/medicina60071145 (PMC11279067; doi:10.3390/medicina60071145)
Supplement: Supplementary file 1 [file medicina-60-01145-s001.zip › medicina-2995511-supplementary/figure_S2.pdf]

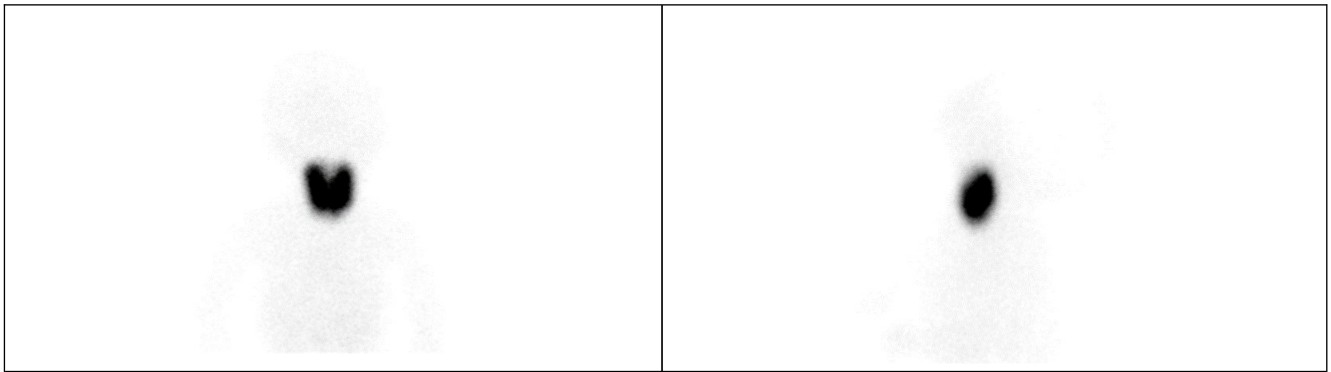

A B

Figure S2. (99m)Tc-pertechnetate thyroid scintigraphy of patient 5. The anteroposterior image (A) and right lateral image (B) show a diffuse goiter with hyperenhancement, increased uptake and enlarged size. The scintigram image for patient 8 was not available.
