# Supplementary material for: Frequency of Mutations in the TPO Gene in Patients with Congenital Hypothyroidism Due to Dyshormonogenesis in Chile
Source: Medicina (Kaunas). 2024 Jul 16;60(7):1145. doi: 10.3390/medicina60071145 (PMC11279067; doi:10.3390/medicina60071145)
Supplement: Supplementary file 1 [file medicina-60-01145-s001.zip › medicina-2995511-supplementary/table_S1.pdf]

Table S1. PCR conditions to amplify each exon

Exon	PCR mixture	PCR thermalcycler setup
1 to 6	0.1uM dNTP, 0.5mM each primer, 1.25U Polimerase[(1)]	94ºC/3 min + (94ºC/30sec + 58ºC/30sec + 72ºC/30sec) x 30 + 72ºC/5min
7, 9, 10, 12	0.1uM dNTP, 0.5mM each primer, 1.25U Polimerase[(1)]	94ºC/3 min + (94ºC/30sec + 58ºC/30sec + 72ºC/30sec) x 35 + 72ºC/5min
11	0.1uM dNTP, 0.25mM each primer, 1.25U Polimerase[(1)]	94ºC/3 min + (94ºC/30sec + 56ºC/30sec + 72ºC/30sec) x 35 + 72ºC/5min
14 and 15	0.1uM dNTP, 0.25mM each primer, 1.25U Polimerase[(1)]	94ºC/3 min + (94ºC/30sec + 58ºC/30sec + 72ºC/30sec) x 35 + 72ºC/5min
16	0.1uM dNTP, 0.25mM each primer, 1.25U Polimerase[(1)]	94ºC/3 min + (94ºC/30sec + 62ºC/30sec + 72ºC/30sec) x 35 + 72ºC/5min
17	0.1uM dNTP, 0.5mM each primer, 1.25U Polimerase[(1)]	94ºC/3 min + (94ºC/30sec + 60ºC/30sec + 72ºC/30sec) x 35 + 72ºC/5min
8.1 and 8.2	0.1uM dNTP, 0.5mM each primer, 0.3uL DMSO, 0.2U Polimerase[(2)]	98ºC/3 min + (98ºC/10sec + 58ºC/10sec + 72ºC/10sec) x 35 + 72ºC/5min

[(1)] Taq DNA Polymerase (RBCBioscience, New Taipei, China), per 10uL PCR reaction
[(2)] Phusion High-Fidelity DNA Polymerase (New England Biolabs, Ipswich, MA, U.S.A.), per 10uL PCR reaction
